# SHFLD3 phenotypes caused by 17p13.3 triplication/ duplication encompassing *Fingerin* (*BHLHA9*) invariably

**DOI:** 10.1186/s13023-022-02480-w

**Published:** 2022-08-26

**Authors:** Ewelina Bukowska-Olech, Anna Sowińska-Seidler, Jolanta Wierzba, Aleksander Jamsheer

**Affiliations:** 1 Department of Medical Genetics, Poznan University of Medical Sciences, Poznan, Poland; 2 Department of Pediatrics and Internal Medicine Nursing, Department of Rare Disorders, Medical University of Gdansk, Gdansk, Poland; 3 Centers for Medical Genetics GENESIS, Poznan, Poland

**Keywords:** Ectrodactyly, Monodactyly, Tibia aplasia, Long bone deficiency, SHFM, Array CGH, Triplication, Duplication, CNV

## Abstract

**Background:**

Split-hand/ foot malformation with long bone deficiency 3 (SHFLD3) is an extremely rare condition associated with duplications located on 17p13.3, which invariably encompasses the *BHLHA9* gene. The disease inherits with variable expressivity and significant incomplete penetrance as high as 50%.

**Results:**

We have detected 17p13.3 locus one-allele triplication in a male proband from family 1 (F1.1), and duplication in a male proband from family 2 (F2.1) applying array comparative genomic hybridization (array CGH). The rearrangements mapped to the following chromosomal regions–arr[GRCh38] 17p13.3(960254–1291856)×4 in F1.1 and arr[GRCh38] 17p13.3(1227482–1302716)×3 in F2.1. The targeted quantitative PCR revealed that the 17p13.3 locus was also duplicated in the second affected member from family 2 (F2.2; brother of F2.1). In the next step, we performed segregation studies using quantitative PCR and revealed that F1.1 inherited the triplication from his healthy father—F1.2, whereas the locus was unremarkable in the mother of F2.1 & F2.2 and the healthy son of F2.1. However, the duplication was present in a healthy daughter of F2.2, an asymptomatic carrier. The breakpoint analysis allowed to define the exact size and span of the duplicated region in Family 2, i.e., 78,948 bp chr17:1225063–1304010 (HG38). Interestingly, all symptomatic carriers from both families presented with variable SHFLD3 phenotype. The involvement of secondary modifying locus could not be excluded, however, the Sanger sequencing screening of *BHLHA9* entire coding sequence was unremarkable for both families.

**Conclusions:**

We have shed light on the one-allele CNV triplication occurrence that should be considered when a higher probe (over duplication range) signal is noted. Second, all SHFLD3 patients were accurately described regarding infrequent limb phenotypes, which were highly variable even when familial. Of note, all symptomatic individuals were males. SHFLD3 still remains a mysterious ultra-rare disease and our findings do not answer crucial questions regarding the disease low penetrance, variable expression and heterogeneity. However, we have presented some clinical and molecular aspects that may be helpful in daily diagnostic routine, both dysmorphological and molecular assessment, of patients affected with SHFLD3.

**Supplementary Information:**

The online version contains supplementary material available at 10.1186/s13023-022-02480-w.

## Introduction

Isolated split-hand/ foot malformation (SHFM), i.e., ectrodactyly, is a heterogeneous group of limb anomalies that manifest either as mild single phalanx hypoplasia or a complete aplasia of phalanges and metatarsals accompanied by additional peripheral skeleton defects, in its most severe form [1]. Following the recent classification proposed by Umair and Hayat, isolated SHFM can be subdivided into type 1, limited to hand or/ and foot (SHFM types 1–6), and type 2 involving long bones deficiencies in addition to hand or/ and foot skeletal deformities (SHFLD types 1–3) [2].

The first cases of split-hand/ foot malformation with long bone deficiency type 3 (SHFLD3; MIM: 612576) were described in a Brazilian family by Richeri-Costa et al. in 1987 as a variant expression of the Gollop-Wolfgang complex (MIM: 228250). Both affected siblings had tibial aplasia-ectrodactyly, whereas one of them also presented with femoral bifurcation [3]. The molecular disease background was revealed almost a quarter of a century later as a variable in size heterozygous duplications in 17p13.3 locus [4]. The critical SHFLD3 region encompasses, however, solely the *BHLHA9* gene (*Fingerin*) encoding a transcription factor involved in embryonic limb development [5]. Besides, recessive point pathogenic variants within the *BHLHA9* are associated with other limb anomalies such as camptosynpolydactyly complex (MIM: 607539) and syndactyly, mesoaxial synostostic, with phalangeal reduction (MIM: 609432) [6, 7].

SHFLD3 occurs in 1 per 1,000,000 live births making it an ultra-rare genetic disease characterized by variable expressivity of phenotypes and incomplete penetrance (as high as 50%). In line with the gnomAD SVs v2.1 database, structural variants located in the *BHLHA9* gene occur with frequency 4.6^e−5^. Interestingly, some researchers have also pointed to a sex bias resulting in a higher ratio of affected males and a higher incidence of having affected male offspring by asymptomatic carriers [5].

## Results

### Clinical report

**Fig. 1:**
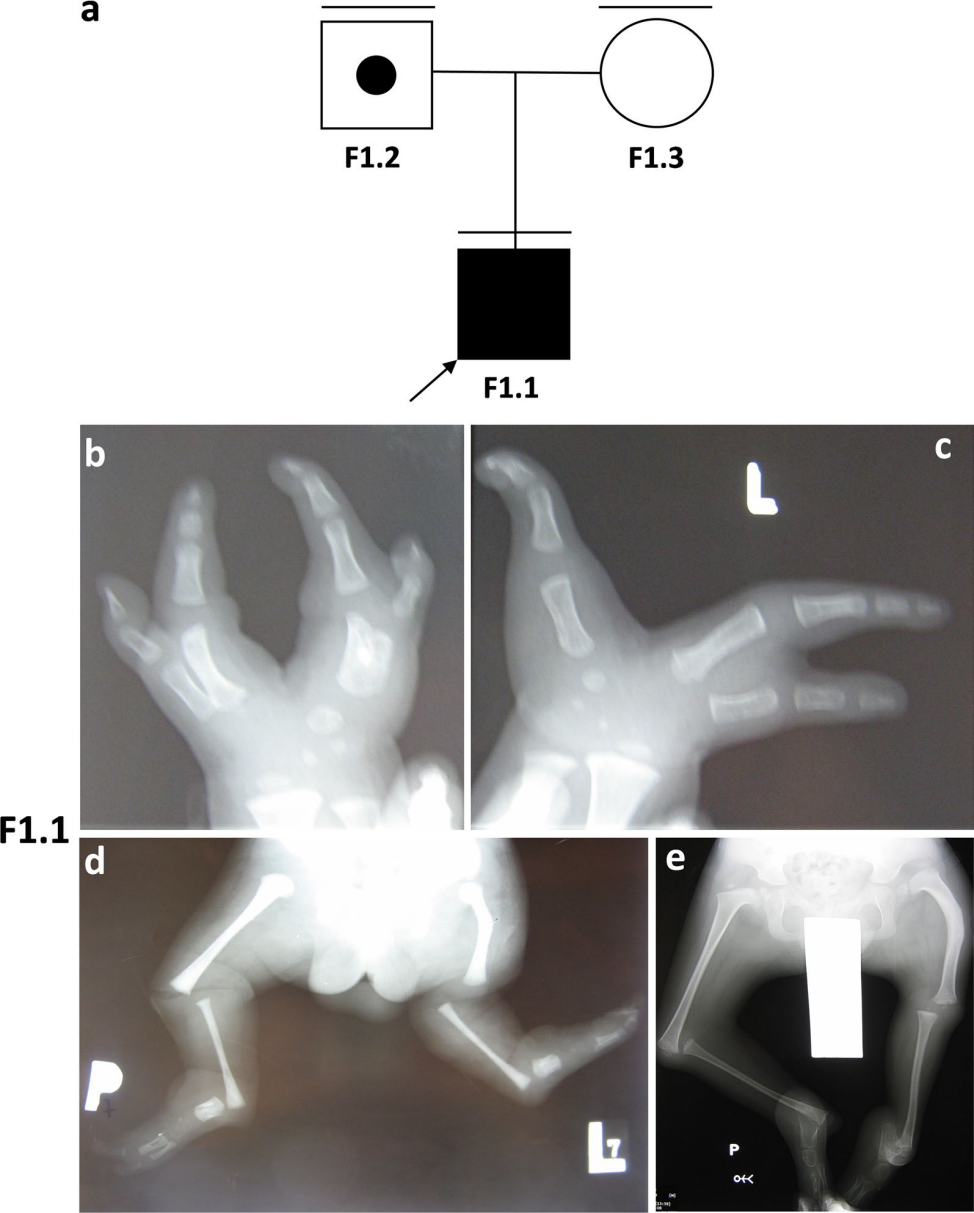
**a** Pedigree of family; F1.1–affected male individual; F1.2–unaffected male carrier of 17p13.3 triplication encompassing *BHLHA9* gene. Results of clinical assessment of individual F1.1. was presented in **b–e**. **b**, **c** Upper extremities X-ray imaging showed hypoplasia of fingers. **d**, **e** Lower extremities X-ray imaging revealed bilateral tibial aplasia and hypoplasia of feet bones. An analysis was made at age of 6 months and 2 years, respectively

**Fig. 2:**
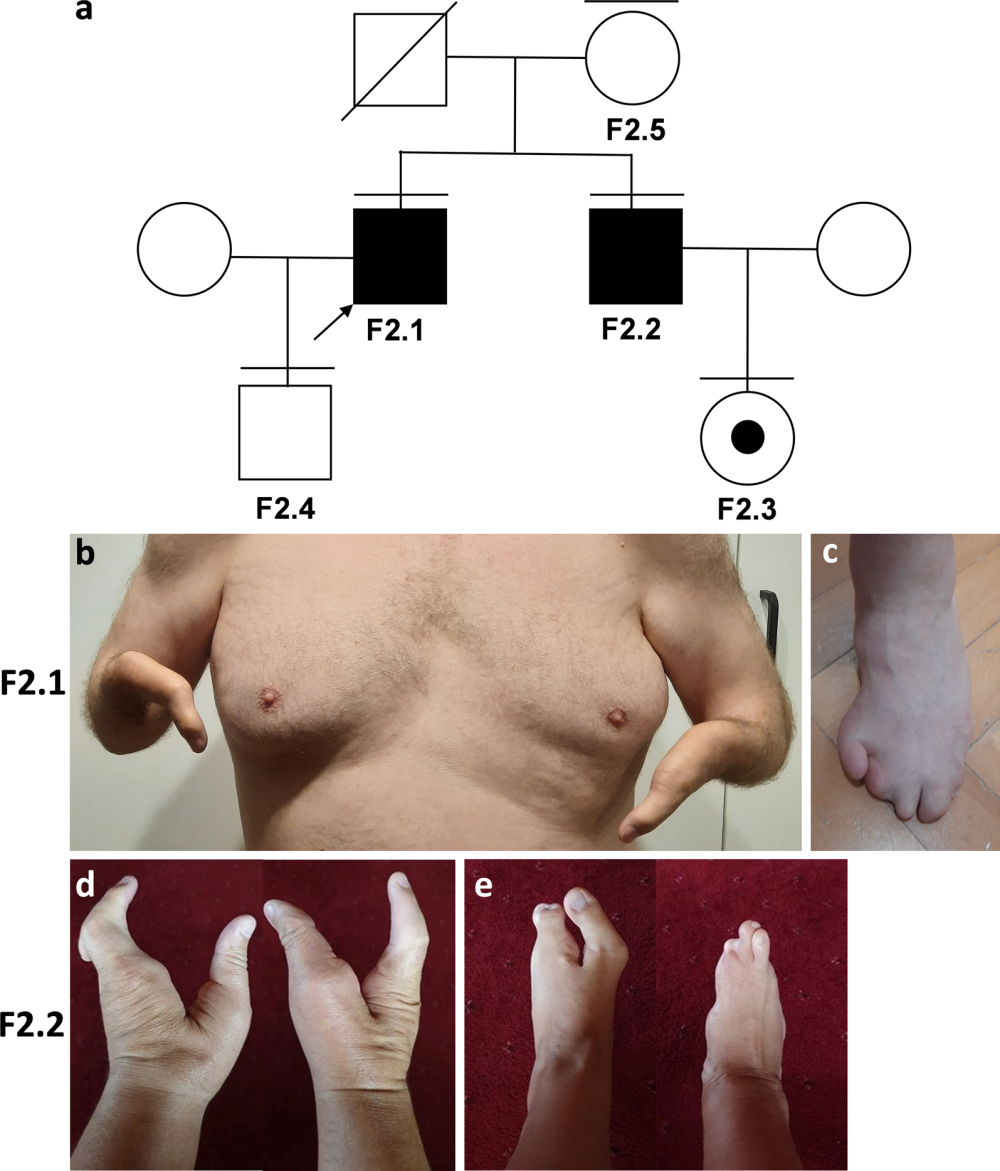
**a** Pedigree of Family 2 has two affected male individuals harboring 17p13.3 duplication encompassing the *BHLHA9* gene—F2.1 & F2.2, and one unaffected female carrier—F2.3. F2.1 **b**, **c** presented with bilateral monodactyly of upper extremities and partial aplasia of the right lower extremity such as femur, tibia, foot aplasia. Note steatomastia. His left foot was hypoplastic. F2.2 **d**, **e** presented bilateral ectrodactyly of upper extremities, left feet ectrodactyly, and hypoplasia of the right foot

A two probands F1.1 (sporadic) and F2.1 (familial) from two different families underwent dysmorphological and molecular analyses due to upper and lower limb abnormalities from the SHFM spectrum (Figs. 1a and 2a). We have noted severe malformations of upper and lower extremities in sporadic individual F1.1 involving bilateral ectrodactyly, bilateral tibial agenesis, and unilateral shortening and campomelic left femur (Fig. 1b–e). Patient F2.1 presented bilateral upper limbs monodactyly, ulnar hypoplasia, severe hypoplasia of the right lower extremity and digital hypoplasia of the left foot (Fig. 2b, c) whereas his brother F2.2 showed divergent phenotype involving bilateral ectrodactyly of upper extremities, unilateral ectrodactyly of the lower left extremity, and absence of digits with almost complete syndactyly of the remaining central toes in the lower right extremity (Fig. 2d, e). The genetic diagnosis was planned according to the SHFM diagnostic algorithm proposed by Sowińska-Seidler et al. [1].

### Molecular analyses

**Fig. 3:**
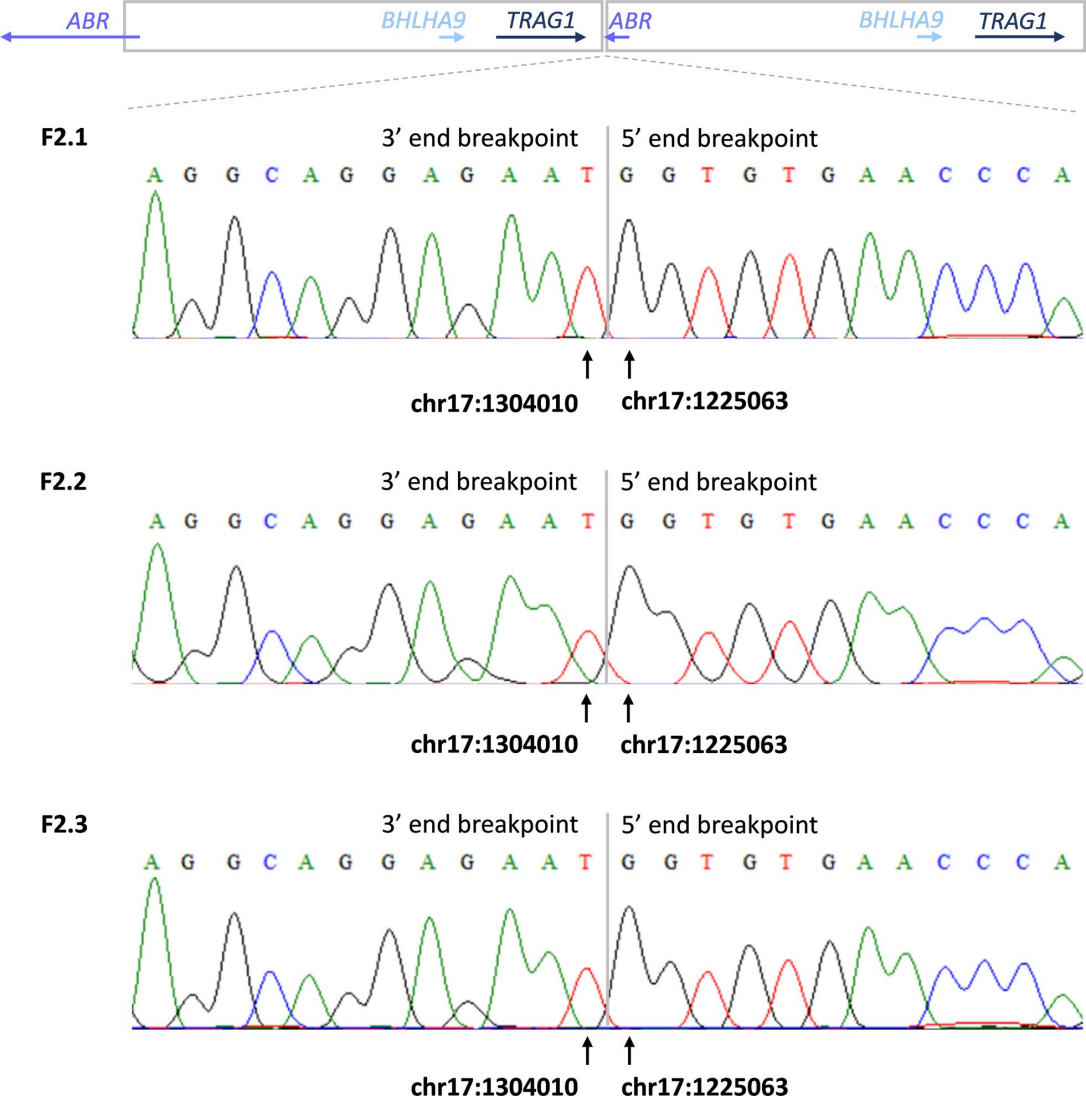
Results of the breakpoint sequencing analysis of the 17p13.3 duplication in members of family 2. We resized the analyzed region by applying a series of quantitative real-time PCR, followed by targeted PCR and Sanger sequencing. The duplication was 78,948 bp in size, resized genomic coordinates–(HG38) 17:1225063–1304010. The same 78,948 bp duplicated region was harbored by affected male individuals F2.1, F2.2, and unaffected female carrier F2.3

Array comparative genomic hybridization (array CGH) had been implemented for F1.1 & F2.1 and has revealed 17p13.3 locus triplication or duplication, respectively. The rearrangements mapped to the following chromosomal regions–arr[GRCh38] 17p13.3(960254–1291856)×4 in F.1.1 and arr[GRCh38] 17p13.3(1227482–1302716)×3 in F2.2. The targeted quantitative PCR revealed that the 17p13.3 locus was also duplicated in the second affected member from family 2 (F2.2; brother of 2.1). In the next step, we had performed segregation studies using quantitative PCR and revealed that F1.1 inherited the triplication from his healthy father—F1.2, whereas the locus was unremarkable in the mother of F2.1 & F2.2 and a healthy son of F2.1. However, the duplication was present in a healthy daughter of F2.2, an asymptomatic carrier. We have also performed a series of quantitative PCRs to narrow down the triplicated or duplicated regions (Additional file 1: Fig. S1 and Additional file 2: Fig. S2). Finally, the breakpoint analysis by Sanger sequencing allowed to define the exact size and span of the duplicated region in Family 2, i.e., 79,948 bp chr17:1225063–1304010 (HG38) (Fig. 3).

Besides, we Sanger sequenced the *BHLHA9* entire coding sequence for either symptomatic (F1.1, F2.1, F2.2) or asymptomatic (F1.2 & F2.3) carriers of duplicated/ triplicated 17p13.3 locus and results of this analysis was unremarkable.

## Discussion

Copy number variations (CNVs) represent a known cause of congenital limb malformations [8, 9]. One example constitutes SHFLD3 linked to a critical duplicated region encompassing one gene–*BHLHA9* [5]. The *BHLHA9*, termed *Fingerin*, is a crucial regulator in limb and finger development that encodes a basic helix-loop-helix (bHLH) A9 transcription factor [10, 11]. It is well known that the gene is dosage-sensitive. Next to gain-of-function mutations resulting in SHFLD3, loss-of-function mutations in *BHLHA9* may cause phenotypes such as camptosynpolydactyly complex (MIM: 607539) and syndactyly, mesoaxial synostostic, with a phalangeal reduction (MIM: 609432) [12].

In the current paper, we have reported two families in which individuals harbor 17p13.3 CNV and, if symptomatic, were diagnosed with SHFLD3. Array CGH 1 × 1 M implemented in F1.1& F2.1 has indicated that the patients harbour the following CNVs–arr[GRCh38] 17p13.3(960254–1291856)×4 and arr[GRCh38] 17p13.3(1227482–1302716)×3, respectively. The chr17:960254–1291856 (HG38) triplication was also present in an unaffected individual F.1.2 (father of F1.1) (Fig. 1a), whereas chr17:1227482–1302716 (HG38) duplication was also carried by an affected F2.2 (brother of F2.1) and an asymptomatic female carrier F2.3 (Fig. 2a). We have excluded the presence of other modifying CNVs in F1.1 and F2.1 along with point mutations within the entire *BHLHA9* coding sequence in all symptomatic and asymptomatic 17p13.3 duplication carriers. Of note, we were unable to exclude the presence of other modifying loci within the genome, however, other researchers performing such studies were unable to find them.

Undeniably, *BHLHA9* triplication was the most interesting finding reported here. So far, only one research has visualized its presence on one chromosome 17, which was Japanese founder triplication detected among five patients with SHFLD3 [13]. To our best knowledge, no other CNVs triplications of one allele were linked to inborn skeletal disorders, or generally, to most Mendelian diseases. However, *SCN1A* triplication was described in Alzheimer's disease, or 1q21.1 triplication was linked to facial dysmorphism, whereas a few aberrations in the form of partial chromosomal triplications were also described applying classical cytogenetic methods [14, 15]. Thus, it may be suggested that one-allele triplications exist with higher frequency but are overlooked applying array CGH methods due to evaluating higher probes signal as regular duplication or using slide formats with improper median probes spacing.

In our cohort, we noted that all affected individuals harboring 17p13.3 copy gain were males. These findings were consistent with the hypothesis claiming the sex bias occurrence [5, 13]. Similar to other SHFLD3 cases, we described highly heterogenous and unique limb phenotypes for a particular patient. Importantly, we did not reveal any changes between phenotype severity and number of 17p13.3 gained copies. For the first time, we have illustrated upper extremities bilateral monodactyly (F2.1), which constituted a severe form of SHFLD3. Reviewing the medical literature, we have noted that bilateral oligodactyly represents a rare clinical feature of SHFM spectrum – sporadically, it was reported in SHFM3 but never in SHFLD3 [16]. Patient F2.1 also had partial hypoplasia of the right lower limb in the form of tibial, foot aplasia, and pes varus, which was operated. On the other hand, his brother–F2.2, had a more “classic” SHFLD3 phenotype as he presented bilateral ectrodactyly of upper extremities, left foot ectrodactyly, and hypoplasia of the right foot. We have listed all clinical features of symptomatic F1.1, F2.1 and F2.2 in Table 1.

**Table 1:** Summary of clinical data observed among symptomatic carriers of 17p13.3 triplication (F1.1) or duplication (F2.1& F2.2) resulting in split hand/foot malformations with long bones deficiencies type 3 (SHFLD3)

	F1.1	F2.1	F2.2
Sex	M	M	M
*Lower extremities*			
Tibial aplasia/ hypoplasia	+	+	−
Distal femoral bifurcation	−	−	−
*Hands*			
Ectrodactyly	+	−	+ (bilateral)
Monodactyly	−	+ (bilateral)	−
Oligodactyly	−	−	−
Brachydactyly	−	−	−
Syndactyly, third and fourth digits	−	−	−
Camptodactyly, third and fourth digits	−	−	−
*Feet*			
Clubfoot	+	−	-
Pes varus	+	+ (operated)	nd
Toe hypoplasia or aplasia	+	+	+
Absent halluces	−	+	−

The list of SHFLD3 features was prepared based on Online Mendelian Inheritance in Man database

+ feature present; − feature absent; *nd* no data

The lack of penetrance of reported here 17p13.3 triplication/ duplication is intriguing. However, lack of 17p13.3 duplication penetrance is a well known phenomenon that was described in all reported so far families [5, 17–19]. One could suspect the presence of additional loci, which modify the SHFLD3 phenotype or block its expression in asymptomatic carriers. Unfortunately, none SHFLD3 locus modifier was discovered so far. Considering the current state of the art, we could suspect the presence of epigenetic changes, i.e., methylation profile changes or histone modifications that have been visualized in some Mendelian disorders recently [20–23]. Unfortunately, we were unable to broaden our research and perform studies to reveal modifying factors/additional genomic or epigenetic changes, whose presence might have explained the asymptomatic 17p13.3 duplication carriers’ occurrence (F1.& F2.3) or phenotypic variability observed in affected male individuals (F1.2, F2.1, F2.2). Another limitation of this study may be the lack of *BHLHA9* expression level analysis, as it is not expressed in available for testing tissues (based on GeneCards database and medical literature review).

## Conclusions

We have shed light on the one-allele CNV triplication occurrence that should be considered when a higher probe (over duplication range) signal is noted. Second, all SHFLD3 patients were accurately described regarding infrequent limb phenotypes, which were highly variable even when familial. Importantly, we have noted the first bilateral monodactyly occurring in SHFLD3. Of note, all symptomatic individuals were males. SHFLD3 still remains a mysterious ultra-rare disease and our findings do not answer crucial questions regarding the disease low penetrance, variable expression and heterogeneity. However, we have presented some clinical and molecular aspects that may be helpful in daily diagnostic routine, both dysmorphological and molecular assessment, of patients affected with SHFLD3.

## Methods

Peripheral blood was collected from affected individuals and their healthy relatives, and next, gDNA was isolated via either the salting-out method or the MagCore^®^ HF16 Automated Nucleic Acid Extractor (RBC Bioscience Corp.).

### Array CGH

We performed array CGH using Agilent Sure Print G3 Human CGH microarray 1 × 1 M kit. The hybridization signals were detected with SureScan Dx Microarray Scanner and visualized using Agilent CytoGenomics software (all from Agilent Technologies) as described previously [24].

### Quantitative PCR (qPCR)

We applied qPCR to determine the 17p13.3 locus dosage and narrow down its genomic coordinates using SYBR dye-based master mix (SYBR Green PCR Master Mix; ThermoFisher Scientific). Reactions ran on the Viia7 cycler as described previously [25]. In short, we applied the comparative 2^−ΔΔCT^ method using control DNA as a calibrator. The results were normalised to albumin gene (*ALB*), whereas factor VIII (*F8*) was targeted in order to assure reliability of the assay (sex determination). Specific primers were designed using the Primer3 tool v. 0.4.0. For primer sequences, see Additional file 3: Table S1.

### PCR and Sanger sequencing

PCR followed by Sanger sequencing were applied to screen for pathogenic mutations in the *BHLHA9* gene in patients F1.1, F1.2, F2.1, F2.2 and F2.3 to establish the exact breakpoints of the duplication in members of Family 2. The PCR reactions and PCR products purification were carried out following standard protocols, whereas specific primers were designed via the Primer3 tool v. 0.4.0. Next, Sanger sequencing was performed on an automated sequencer Applied Biosystems Prism 3700 DNA Analyzer using dye-terminator chemistry kit v.3, ABI 3130XL [26]. The analysis was performed applying the BioEdit tool and annotated against the GRCh38 human reference genome to map the deletion breakpoints or reference sequence NM_001164405.2 to analyze the *BHLHA9* coding region. The reaction conditions are available upon request. For primer sequences, see Additional file 4: Table S2.

## Supplementary Information

**Additional file 1: Fig. S1** Results of copy number analysis in 17p13.3 locus in Family 1. Segregation studies in Family 1. We performed quantitative real-time PCR on DNA from all individuals from Family 1 (F1.1–affected male individual; F1.2–unaffected male carrier; F1.3–healthy mother of F1.1) and one unrelated control. DNA quantity from 17p13.3 locus and flanking regions was compared to 2 reference genes (*ALB* and *F8*) using the comparative 2^−ΔΔCT^ quantification method (**a**). In addition, we narrowed down the 17p13.3 duplication and triplication regions for F1.1 (**b**). Error bars represent standard deviation. Ratios can be interpreted as follows: normal (0.9–1.1), one-allele duplication (1.4–1.6), one-allele triplication (1.8–2.4)

**Additional file 2: Fig. S2** Results of segregation studies in Family 2. We performed quantitative real-time PCR on DNA from all individuals from Family 2 (F2.1, F2.2–affected male individuals; F2.3–unaffected female carrier; F2.4 & F2.5–healthy individuals) and one unrelated control. DNA quantity from 17p13.3 locus and flanking regions was compared to 2 reference genes (*ALB *and *F8*) using the comparative 2^−ΔΔCT^ quantification method. Error bars represent standard deviation. Ratios can be interpreted as normal (0.65–1.0) and one-allele duplication (1.4–1.5)

**Additional file 3: Table S1** Oligonucleotide primers used to perform copy number analysis of the 17p13.3 region

**Additional file 4: Table S2** Oligonucleotide primers used to perform PCR and Sanger sequencing

## Data Availability

The datasets for this article are not publicly available due to concerns regarding participants’/patients’ anonymity. Requests to access the datasets should be directed to the corresponding authors.

## Abbreviations

Array CGH: Array comparative genomic hybridization
CNV: Copy number variations
SHFLD3: Split-hand/foot malformation with long bone deficiency type 3
SHFM: Split-hand/foot malformation

## References

[1] Sowińska-Seidler A, Socha M, Jamsheer A (2014). Split-hand/foot malformation-molecular cause and implications in genetic counseling. J Appl Genet.

[2] Umair M, Hayat A (2019). Nonsyndromic split-hand/foot malformation: recent classification. Mol Syndromol.

[3] Richieri-Costa A, Brunoni D, Laredo Filho J, Kasinski S (1987). Tibial aplasia-ectrodactyly as variant expression of the Gollop-Wolfgang complex: report of a Brazilian family. Am J Med Genet.

[4] Armour CM, Bulman DE, Jarinova O, Rogers RC, Clarkson KB, DuPont BR, Dwivedi A, Bartel FO, McDonell L, Schwartz CE, Boycott KM, Everman DB, Graham GE (2011). 17p13.3 microduplications are associated with split-hand/foot malformation and long-bone deficiency (SHFLD). Eur J Hum Genet EJHG.

[5] Klopocki E, Lohan S, Doelken SC, Stricker S, Ockeloen CW, Thiele S, de Aguiar R, Lezirovitz K, Mingroni Netto RC, Jamsheer A, Shah H, Kurth I, Habenicht R, Warman M, Devriendt K, Kordass U, Hempel M, Rajab A, Mäkitie O, Naveed M, Radhakrishna U, Antonarakis SE, Horn D, Mundlos S (2012). Duplications of BHLHA9 are associated with ectrodactyly and tibia hemimelia inherited in non-Mendelian fashion. J Med Genet.

[6] Malik S, Percin FE, Bornholdt D, Albrecht B, Percesepe A, Koch MC, Landi A, Fritz B, Khan R, Mumtaz S, Akarsu NA, Grzeschik KH (2014). Mutations affecting the BHLHA9 DNA-binding domain cause MSSD, mesoaxial synostotic syndactyly with phalangeal reduction, malik-percin type. Am J Hum Genet.

[7] Phadke SR, Kar A, Das BA, Dalal A (2016). Complex Camptosynpolydactyly and Mesoaxial synostotic syndactyly with phalangeal reduction are allelic disorders. Am J Med Genet A.

[8] Alvarado DM, Buchan JG, Frick SL, Herzenberg JE, Dobbs MB, Gurnett CA (2013). Copy number analysis of 413 isolated talipes equinovarus patients suggests role for transcriptional regulators of early limb development. Eur J Hum Genet EJHG.

[9] Hilger AC, Dworschak GC, Reutter HM (2020). Lessons learned from CNV analysis of major birth defects. Int J Mol Sci.

[10] Kataoka K, Matsushima T, Ito Y, Sato T, Yokoyama S, Asahara H (2018). Bhlha9 regulates apical ectodermal ridge formation during limb development. J Bone Miner Metab.

[11] Schatz O, Langer E, Ben-Arie N, Montero JA, Lorda-Diez CI, Sanchez-Fernandez C, Hurle JM (2014). Cell death in the developing vertebrate limb: a locally regulated mechanism contributing to musculoskeletal tissue morphogenesis and differentiation. Dev Dyn Off Publ Am Assoc Anat.

[12] Schatz O, Langer E, Ben-Arie N (2014). Gene dosage of the transcription factor Fingerin (bHLHA9) affects digit development and links syndactyly to ectrodactyly. Hum Mol Genet.

[13] Nagata E, Kano H, Kato F, Yamaguchi R, Nakashima S, Takayama S, Kosaki R, Tonoki H, Mizuno S, Watanabe S, Yoshiura K, Kosho T, Hasegawa T, Kimizuka M, Suzuki A, Shimizu K, Ohashi H, Haga N, Numabe H, Horii E, Nagai T, Yoshihashi H, Nishimura G, Toda T, Takada S, Yokoyama S, Asahara H, Sano S, Fukami M, Ikegawa S, Ogata T (2014). Japanese founder duplications/triplications involving BHLHA9 are associated with split-hand/foot malformation with or without long bone deficiency and Gollop-Wolfgang complex. Orphanet J Rare Dis.

[14] Zafar F, Valappil RA, Kim S, Johansen KK, Chang ALS, Tetrud JW, Eis PS, Hatchwell E, Langston JW, Dickson DW, Schüle B (2018). Genetic fine-mapping of the Iowan SNCA gene triplication in a patient with Parkinson’s disease. NPJ Parkinson’s Dis.

[15] Van Dijck A, van der Werf IM, Reyniers E, Scheers S, Azage M, Siefkas K, Van der Aa N, Lacroix A, Rosenfeld J, Argiropoulos B, Davis K, Innes AM, Mefford HC, Mortier G, Meuwissen M, Kooy RF (2015). Five patients with a chromosome 1q21.1 triplication show macrocephaly, increased weight and facial similarities. Eur J Med Genet.

[16] Holder-Espinasse M, Jamsheer A, Escande F, Andrieux J, Petit F, Sowinska-Seidler A, Socha M, Jakubiuk-Tomaszuk A, Gerard M, Mathieu-Dramard M, Cormier-Daire V, Verloes A, Toutain A, Plessis G, Jonveaux P, Baumann C, David A, Farra C, Colin E, Jacquemont S, Rossi A, Mansour S, Ghali N, Moncla A, Lahiri N, Hurst J, Pollina E, Patch C, Ahn JW, Valat A-S, Mezel A, Bourgeot P, Zhang D, Manouvrier-Hanu S (2019). Duplication of 10q24 locus: broadening the clinical and radiological spectrum. Eur J Hum Genet EJHG.

[17] Shen Y, Si N, Liu Z, Liu F, Meng X, Zhang Y, Zhang X (2018). 17p13.3 genomic rearrangement in a Chinese family with split-hand/foot malformation with long bone deficiency: report of a complicated duplication with marked variation in phenotype. Orphanet J Rare Dis.

[18] Paththinige CS, Sirisena ND, Escande F, Manouvrier S, Petit F, Dissanayake VHW. Split hand/foot malformation with long bone deficiency associated with BHLHA9 gene duplication: a case report and review of literature. BMC Med Genet. 2019;20. 10.1186/s12881-019-0839-2 PMC6570964 31200655

[19] Fusco C, De NP, Alfaiz AA, Pellico MT, Augello B, Malerba N, Zelante L, Reymond A, Merla G (2017). A new split hand/foot malformation with long bone deficiency familial case. J Pediatric Genet.

[20] Fahrner JA, Bjornsson HT (2019). Mendelian disorders of the epigenetic machinery: postnatal malleability and therapeutic prospects. Hum Mol Genet.

[21] Kim J-H, Lee JH, Lee I-S, Lee SB, Cho KS (2017). Histone lysine methylation and neurodevelopmental disorders. Int J Mol Sci.

[22] Beck DB, Petracovici A, He C, Moore HW, Louie RJ, Ansar M, Douzgou S, Sithambaram S, Cottrell T, Santos-Cortez RLP, Prijoles EJ, Bend R, Keren B, Mignot C, Nougues M-C, Õunap K, Reimand T, Pajusalu S, Zahid M, Saqib MAN, Buratti J, Seaby EG, McWalter K, Telegrafi A, Baldridge D, Shinawi M, Leal SM, Schaefer GB, Stevenson RE, Banka S, Bonasio R, Fahrner JA (2020). Delineation of a human mendelian disorder of the DNA demethylation machinery: TET3 deficiency. Am J Hum Genet.

[23] Levy MA, Beck DB, Metcalfe K, Douzgou S, Sithambaram S, Cottrell T, Ansar M, Kerkhof J, Mignot C, Nougues M-C, Keren B, Moore HW, Oegema R, Giltay JC, Simon M, van Jaarsveld RH, Bos J, van Haelst M, Motazacker MM, Boon EMJ, Santen GWE, Ruivenkamp CAL, Alders M, Luperchio TR, Boukas L, Ramsey K, Narayanan V, Schaefer GB, Bonasio R, Doheny KF, Stevenson RE, Banka S, Sadikovic B, Fahrner JA (2021). Deficiency of TET3 leads to a genome-wide DNA hypermethylation episignature in human whole blood. NPJ Genomic Med.

[24] Bukowska-Olech E, Dmitrzak-Węglarz M, Larysz D, Simon D, Walczak-Sztulpa JJA (2020). Compound craniosynostosis, intellectual disability, and Noonan-like facial dysmorphism associated with 7q32. 3–q35 deletion. Birth Defects Res.

[25] Walczak-Sztulpa J, Posmyk R, Bukowska-Olech EM, Wawrocka A, Jamsheer A, Oud MM, Schmidts M, Arts HH, Latos-Bielenska A, Wasilewska A (2020). Compound heterozygous IFT140 variants in two Polish families with Sensenbrenner syndrome and early onset end-stage renal disease. Orphanet J Rare Dis.

[26] Bukowska-Olech E, Sowińska-Seidler A, Łojek F, Popiel D, Walczak-Sztulpa J, Jamsheer A (2020). Further phenotypic delineation of the auriculocondylar syndrome type 2 with literature review. J Appl Genet.

