# Supplementary figures and images for: SHFLD3 phenotypes caused by 17p13.3 triplication/ duplication encompassing Fingerin (BHLHA9) invariably
Source: Orphanet J Rare Dis. 2022 Aug 26;17:325. doi: 10.1186/s13023-022-02480-w (PMC9419377; doi:10.1186/s13023-022-02480-w)

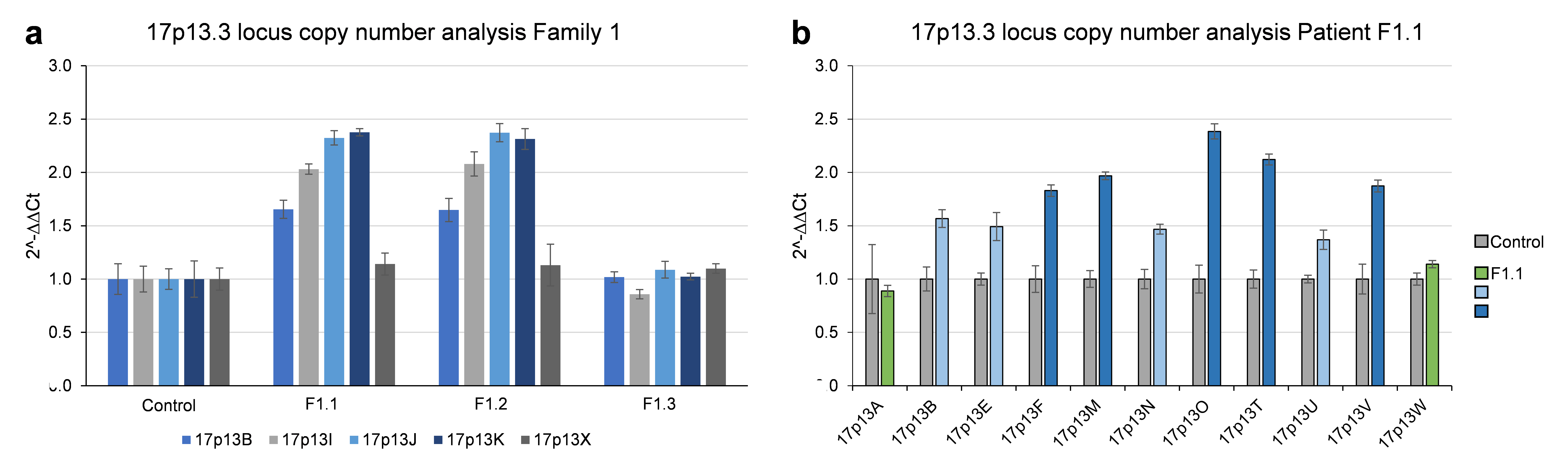

Supplement: Supplementary file 1 — Additional file 1: Fig. S1 Results of copy number analysis in 17p13.3 locus in Family 1. Segregation studies in Family 1. We performed quantitative real-time PCR on DNA from all individuals from Family 1 (F1.1–affected male individual; F1.2–unaffected male carrier; F1.3–healthy mother of F1.1) and one unrelated control. DNA quantity from 17p13.3 locus and flanking regions was compared to 2 reference genes (ALB and F8) using the comparative 2−ΔΔCT quantification method (a). In addition, we narrowed down the 17p13.3 duplication and triplication regions for F1.1 (b). Error bars represent standard deviation. Ratios can be interpreted as follows: normal (0.9–1.1), one-allele duplication (1.4–1.6), one-allele triplication (1.8–2.4) [file 13023_2022_2480_MOESM1_ESM.tif]

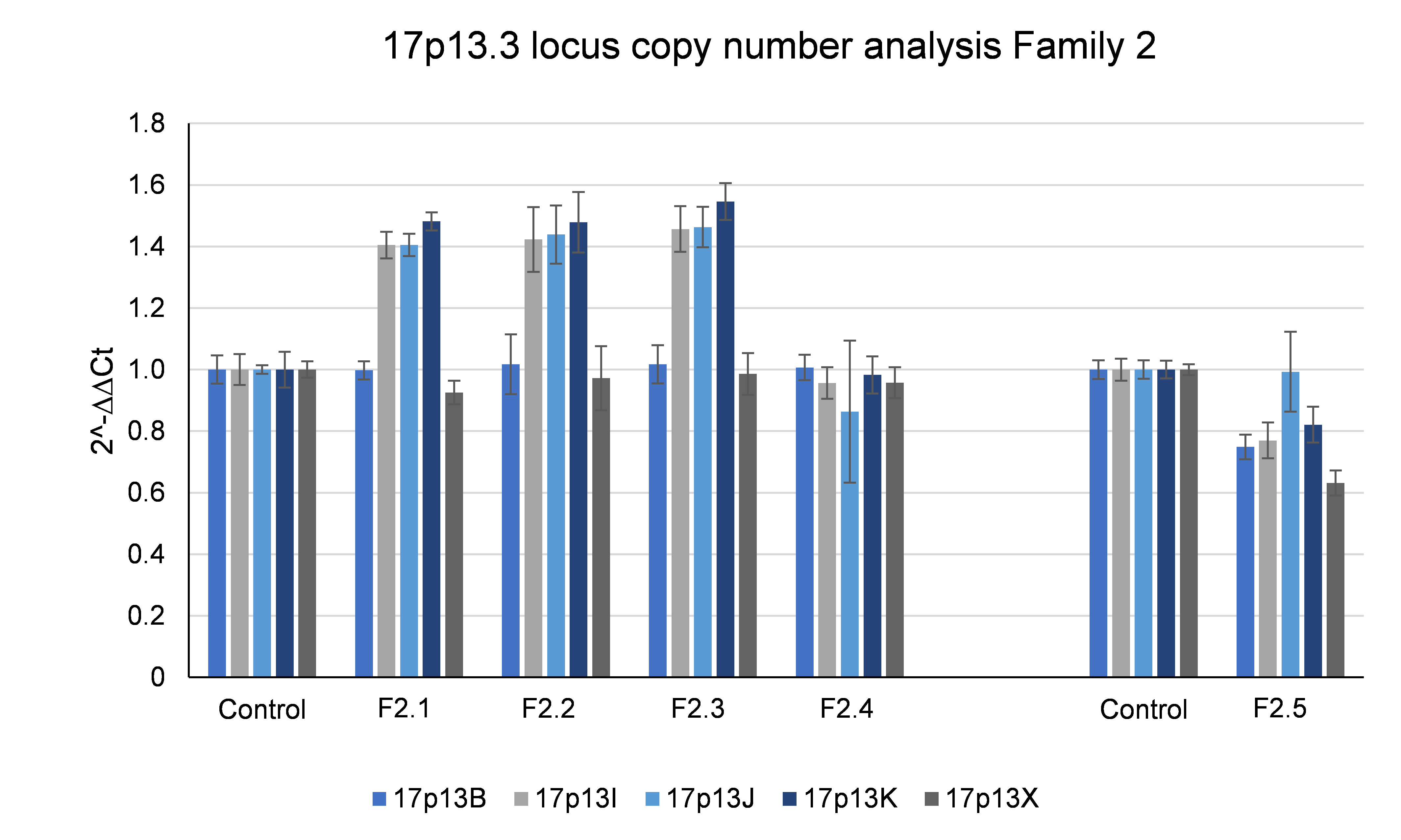

Supplement: Supplementary file 2 — Additional file 2: Fig. S2 Results of segregation studies in Family 2. We performed quantitative real-time PCR on DNA from all individuals from Family 2 (F2.1, F2.2–affected male individuals; F2.3–unaffected female carrier; F2.4 & F2.5–healthy individuals) and one unrelated control. DNA quantity from 17p13.3 locus and flanking regions was compared to 2 reference genes (ALB and F8) using the comparative 2−ΔΔCT quantification method. Error bars represent standard deviation. Ratios can be interpreted as normal (0.65–1.0) and one-allele duplication (1.4–1.5) [file 13023_2022_2480_MOESM2_ESM.tif]
