# Supplementary material for: SHFLD3 phenotypes caused by 17p13.3 triplication/ duplication encompassing Fingerin (BHLHA9) invariably
Source: Orphanet J Rare Dis. 2022 Aug 26;17:325. doi: 10.1186/s13023-022-02480-w (PMC9419377; doi:10.1186/s13023-022-02480-w)
Supplement: Supplementary file 3 — Additional file 3: Table S1 Oligonucleotide primers used to perform copy number analysis of the 17p13.3 region [file 13023_2022_2480_MOESM3_ESM.docx]

**Additional file 3: Table S1** Oligonucleotide primers used to perform copy number analysis of the 17p13.3 region.

| **Primer name** | **sequence (5'->3')** | **Genomic coordinates (hg38)** | **Target** | **Copy number** |
| --- | --- | --- | --- | --- |
| **Reference gene** | | | | |
| ALB_F | TGAAATGGCTGACTGCTGTG | chr4:73408650-73408732 | Albumin (*ALB*) | Normal |
| ALB_R | GGAGGTTTGGGTTGTCATCT |  |  |  |
| **Sex determination** | | | | |
| F8_F | TTTCCATTCAACACCTCAGTCGT | chrX:154999491-154999575 | Factor VIII (*F8*) | Normal |
| F8_R | GCCTTGGCTTAGCGATGTTG |  |  |  |
| **Family 1** | | | | |
| 17p13_AF | TTTCTCTGCCCTCAGGAAAC | chr17:956311-956397 | 17p13.3 | Normal |
| 17p13_AR | ATAGTGCTGGGCACAGAGGA |  |  |  |
| 17p13_BF | CACAGAAATCACACCTCCAC | chr17:957883-957971 |  | Duplication |
| 17p13_BR | ACGTTCTGTGAGGAAAACAG |  |  |  |
| 17p13_CF | CTAGGATCTCGGCAGGACAC | chr17:970304-970393 |  |  |
| 17p13_CR | CCCCTTCATCCTACGGAAAG |  |  |  |
| 17p13_DF | CTGGCTGGACACTGACTCCT | chr17:980348-980434 |  |  |
| 17p13_DR | CTAACCAGACCCCACCAGAA |  |  |  |
| 17p13_EF | GGTGTGGAAAGAGGGGATTT | chr17:984161-984245 |  |  |
| 17p13_ER | CAAATGACTCCTTGGGCTTC |  |  |  |
| 17p13_FF | CGTGACCTTATGCTCTGCTG | chr17:986560-986639 |  | Triplication |
| 17p13_FR | GCACCACTGTTGGGTCTTCT |  |  |  |
| 17p13_GF | GGCAGCATTTTGTCCTCACT | chr17:987043-987132 |  |  |
| 17p13_GR | GGATGCTAGTTTTGGCAGGA |  |  |  |
| 17p13_HF | TGGGTCAGAGAACCTCCAGT | chr17:988484-988572 |  |  |
| 17p13_HR | GCCTTCTTGGAGACTGATGC |  |  |  |
| 17p13_IF | GGGAACTCATTCATGGCAAG | chr17:1271936-1272020 |  |  |
| 17p13_IR | GGAGGGGTCTCTGGATGTCT |  |  |  |
| 17p13_JF | TGTGTAGCCTCCGTTACGTG | chr17:1274114-1274195 |  |  |
| 17p13_JR | ATGGACACGATCTTGGGAAG |  |  |  |
| 17p13_KF | AGAAGCCCCCAGAGATTACC | chr17:1280295-1280378 |  |  |
| 17p13_KR | ATGATGAGGGGGATGAGGTT |  |  |  |
| 17p13_LF | GCTCCACAGCTACGCTGACT | chr17:1280992-1281078 |  |  |
| 17p13_LR | CATGCCCTCTGACTCTCACA |  |  |  |
| 17p13_MF | CTCAATGCCTGCACGAGAG | chr17:1283232-1283312 |  |  |
| 17p13_MR | ACCCTCCTGGTGTTGTTCTG |  |  |  |
| 17p13_NF | TTTGCATGTCGTCTTCAAGC | chr17:1284313-1284399 |  | Duplication |
| 17p13_NR | ATCCAACCACACTCCTCTCG |  |  |  |
| 17p13_OF | GTGGGTTGTACCGACCATTC | chr17:1284563-1284652 |  | Triplication |
| 17p13_OR | GGCCTGACCGTCCTCTACTA |  |  |  |
| 17p13_PF | CAGCAGGGACTTGCTTAGGA | chr17:1285100-1285187 |  |  |
| 17p13_PR | TCCACTTGTTGTCTCCCAAA |  |  |  |
| 17p13_QF | TCCACCCCGTACTCTACACA | chr17:1290160-1290248 |  |  |
| 17p13_QR | CAGGGAGCCAGTGAAGGATA |  |  |  |
| 17p13_RF | TGGCAGAATTTGGCATCATA | chr17:1291651-1291740 |  |  |
| 17p13_RR | CAAGATTCCTCCAGCTCTGC |  |  |  |
| 17p13_SF | TGCATTTGGACTTCGTTCTG | chr17:1292229-1292312 |  |  |
| 17p13_SR | GCTGTTGCAAAGGTTCAGTG |  |  |  |
| 17p13_TF | CTCCTGTGCCCACAGAACTT | chr17:1292786-1292866 |  |  |
| 17p13_TR | ACCCACCACAGTTGCATTTT |  |  |  |
| 17p13_UF | ACGGAGCTTGGAATGGAAGT | chr17:1293749-1293829 |  | Duplication |
| 17p13_UR | CCTCTTGGGGTCCTGTTCTC |  |  |  |
| 17p13_VF | CACTGTCTTCACAGCCTCCA | chr17:1294387-1294466 |  | Triplication |
| 17p13_VR | CTTTTGTGTGATCCGGGAAG |  |  |  |
| 17p13_WF | GACCTGGTCCACCTTCTCAG | chr17:1295162-1295244 |  | Normal |
| 17p13_WR | AAGCCTGGCACTTCTTCTCA |  |  |  |
| 17p13_XF | AACCATTCCGCCATTTATCC | chr17:1337484-1337573 |  |  |
| 17p13_XR | CCATGACAAATAAAGCACCTGT |  |  |  |
| **Family 2** | | | | |
| 17p13_BF | CACAGAAATCACACCTCCAC | chr17:957883-957971 | 17p13.3 | Normal |
| 17p13_BR | ACGTTCTGTGAGGAAAACAG |  |  |  |
| 17p13_A1F | GGCCGAGAGTATGGTGACTC | chr17:1221413-1221494 |  |  |
| 17p13_A1R | GGAGGTGAGCTTGAGGACAG |  |  |  |
| 17p13_B1F | AAGGATTGAATTTTCAGCCTCA | chr17:1222365-1222450 |  |  |
| 17p13_B1R | GGCCTCAAATGGCTTCTTAAT |  |  |  |
| 17p13_C1F | AAGGCAAAGGAAAAGCAACC | chr17:1223728-1223817 |  |  |
| 17p13_C1R | GGTGGTTTTAAAGCGTTTGG |  |  |  |
| 17p13_D1F | AGCCATTGTACCAGCCACAT | chr17:1225281-1225363 |  | Duplication |
| 17p13_D1R | TTCCTCAGAGCTTCCCACTC |  |  |  |
| 17p13_E1F | CCCCAGCTCACGTCTGTAGT | chr17:1227072-1227154 |  |  |
| 17p13_E1R | GGGAATTGCAAAAGATGCTC |  |  |  |
| 17p13_IF | GGGAACTCATTCATGGCAAG | chr17:1271936-1272020 |  |  |
| 17p13_IR | GGAGGGGTCTCTGGATGTCT |  |  |  |
| 17p13_JF | TGTGTAGCCTCCGTTACGTG | chr17:1274114-1274195 |  |  |
| 17p13_JR | ATGGACACGATCTTGGGAAG |  |  |  |
| 17p13_KF | AGAAGCCCCCAGAGATTACC | chr17:1280295-1280378 |  |  |
| 17p13_KR | ATGATGAGGGGGATGAGGTT |  |  |  |
| 17p13_F1F | AGGTGAAATGCCACCAGTGT | chr17:1302494-1302573 |  |  |
| 17p13_F1R | TCGCTGACAGAGGGTGTGTA |  |  |  |
| 17p13_G1F | GGGTGCCACTGCTAGTGAAT | chr17:1303541-1303622 |  |  |
| 17p13_G1R | GGATCACCTGGAAGGCTTTT |  |  |  |
| 17p13_H1F | GGGTCTCACAGCAGAAGAGG | chr17:1304428-1304516 |  | Normal |
| 17p13_H1R | CCCACACAAAGGTGGTCTTC |  |  |  |
| 17p13_I1F | AGAGCGAGGAAGCACAGAAG | chr17:1305134-1305220 |  |  |
| 17p13_I1R | TAAGCAAAGAGGAGCCAACC |  |  |  |
| 17p13_XF | AACCATTCCGCCATTTATCC | chr17:1337484-1337573 |  |  |
| 17p13_XR | CCATGACAAATAAAGCACCTGT |  |  |  |
