# Supplementary material for: SHFLD3 phenotypes caused by 17p13.3 triplication/ duplication encompassing Fingerin (BHLHA9) invariably
Source: Orphanet J Rare Dis. 2022 Aug 26;17:325. doi: 10.1186/s13023-022-02480-w (PMC9419377; doi:10.1186/s13023-022-02480-w)
Supplement: Supplementary file 4 — Additional file 4: Table S2 Oligonucleotide primers used to perform PCR and Sanger sequencing [file 13023_2022_2480_MOESM4_ESM.docx]

**Additional file 4: Table S2** Oligonucleotide primers used to perform PCR and Sanger sequencing

| **Primer name** | **sequence (5'->3')** | **Genomic coordinates (hg38)** | **Target** |
| --- | --- | --- | --- |
| **sequencing of *BHLHA9* coding region** | | | |
| BHLHA9e1aF | ATAAAGCCCAGCTGGAAGG | chr17:1270415-1270975 | *BHLHA9* |
| BHLHA9e1aR | CGTGGCACTCCAGGTGTC |  |  |
| BHLHA9e1bF | CTACAACGAGGCCTTCAACG | chr17:1270803-1271342 |  |
| BHLHA9e1bR | TGCAGTCCCGGGTTTTATAG |  |  |
| **17p13.3 duplication breakpoint sequencing in Family 2** | | | |
| 17p13_G1F | GGGTGCCACTGCTAGTGAAT | chr17:1303541 | Mapping the breakpoints |
| 17p13_D1R | TTCCTCAGAGCTTCCCACTC | chr17: 1225363 |  |
